# The mediating role of sleep quality in the relationship between professional identity and anxiety symptoms among emergency nurses: a cross-sectional study

**DOI:** 10.3389/fpubh.2026.1754599

**Published:** 2026-03-17

**Authors:** Siyu Cao, Hong Li, Tao Cheng

**Affiliations:** 1 Emergency Department of West China Hospital, Sichuan University/West China School of Nursing, Sichuan University, Chengdu, China; 2 Disaster Medical Center, Sichuan University, Chengdu, China; 3 Nursing Key Laboratory of Sichuan Province, Chengdu, China

**Keywords:** anxiety, cross-sectional study, emergency nurses, mediation, professional identity, sleep quality

## Abstract

**Background:**

Emergency nurses have long been exposed to high-intensity workloads and psychologically demanding environments, placing them at increased risk of anxiety symptoms. Professional identity is considered an important occupational resource that may buffer psychological distress, yet little is known about the mechanisms through which it influences anxiety. Sleep quality, a crucial determinant of mental health, may serve as a potential mediator in this association.

**Objective:**

This study aimed to examine the relationship between professional identity and anxiety symptoms among emergency nurses and to explore whether sleep quality mediates this association.

**Methods:**

A cross-sectional study was conducted among 179 emergency nurses from a tertiary teaching hospital in China. Participants completed the Generalized Anxiety Disorder Scale (GAD-7), the Pittsburgh Sleep Quality Index (PSQI), and the Professional Identity Scale for Nurses (PISN). Based on a GAD-7 cutoff score of ≥5, participants were classified into the anxiety group (*n* = 69, 38.5%) and the non-anxiety group (*n* = 110, 61.5%). Between-group comparisons were conducted using Mann–Whitney *U* and Chi-square tests. Correlations were assessed using Spearman coefficients. Hierarchical regression was performed to identify predictors of anxiety, and a mediation analysis was conducted using a bias-corrected bootstrap approach.

**Results:**

The prevalence of anxiety symptoms was 38.5%. Nurses with anxiety showed significantly poorer sleep quality and lower professional identity levels compared with non-anxious nurses (*p* < 0.01). Professional identity was negatively correlated with both PSQI scores (*r* = −0.31, *p* < 0.001) and anxiety symptoms (*r* = −0.27, *p* < 0.001). Hierarchical regression indicated that poorer sleep quality (β = 0.32, *p* < 0.001) and lower professional identity (β = −0.15, *p* = 0.003) were significant predictors of anxiety symptoms. Mediation analysis revealed that sleep quality partially mediated the effect of professional identity on anxiety (indirect effect = −0.06, 95% CI: −0.10 to −0.02), accounting for approximately 28.6% of the total effect.

**Conclusion:**

Lower professional identity and poor sleep quality are significant risk factors for anxiety symptoms among emergency nurses. Sleep quality plays a partial mediating role in this association. Interventions designed to strengthen professional identity while improving sleep health may effectively reduce anxiety symptoms in this workforce.

## 1 Introduction

Emergency departments operate under conditions characterized by unpredictable workloads, time pressure, overcrowding, and constant exposure to critical and traumatic events. Recent studies published between 2024 and 2025 consistently demonstrate that emergency nurses experience significantly higher levels of psychological distress—particularly anxiety—than nurses in other clinical settings, underscoring the persistence and severity of this problem in contemporary practice (1, 2). Anxiety not only affects the wellbeing of nurses but may also impair clinical judgment, decision-making, and patient safety (3). Understanding the factors associated with anxiety in this workforce is therefore essential for guiding effective interventions.

Professional identity is a core component of nurses' professional development and motivation. It reflects individuals' understanding, internalization, and valuation of their professional role. A strong professional identity has been associated with higher job satisfaction, stronger organizational commitment, and better psychological resilience (4, 5). Recent empirical evidence indicates that higher professional identity is associated with reduced negative emotional responses under clinical stress, even in high-demand training and practice contexts (6, 7). However, the pathways through which professional identity influences psychological outcomes remain insufficiently understood, especially within the high-pressure emergency care environment (8).

Sleep disturbance is another critical factor closely linked to mental health among nurses. Shift work, circadian disruption, and occupational stress commonly lead to poor sleep quality in emergency nurses (9). A growing body of recent research demonstrates not only that poor sleep quality is strongly associated with anxiety, but also that sleep disturbance represents one of the most robust and clinically actionable correlates of psychological distress among shift-working nurses. At the same time, emerging research suggests that sleep may serve as a mechanism linking occupational stressors or resources to psychological outcomes (10, 11). Whether sleep quality mediates the relationship between professional identity and anxiety in emergency nurses has not been examined.

While the individual links between professional identity, sleep quality, and anxiety are plausible, an integrated model examining their interrelationships is lacking. It is possible that a strong professional identity serves as a buffer, fostering better psychological adaptation that protects sleep quality, thereby reducing anxiety. Alternatively, the erosion of professional identity under constant stress may exacerbate sleep problems, which subsequently contribute to heightened anxiety. To our knowledge, few studies have examined whether sleep quality mediates the relationship between professional identity and anxiety symptoms among emergency nurses (10, 12, 13).

To address these gaps, this study aimed to (1) examine the levels of professional identity, sleep quality, and anxiety among emergency nurses; (2) analyze the associations among these variables; and (3) explore whether sleep quality mediates the relationship between professional identity and anxiety. Clarifying this mechanism may provide theoretical support for designing interventions that enhance psychological wellbeing and promote sustainable workforce development in emergency nursing. Although this study uses a conventional analytic approach, it is, to our knowledge, few studies have integrated professional identity, sleep quality, and anxiety into a single model among emergency nurses. Given the cross-sectional design, the mediation analysis can only indicate statistical associations rather than causal relationships, and the model should be interpreted as reflecting indirect associations in the data rather than confirmed causal pathways.

## 2 Materials and methods

### 2.1 Study design

This cross-sectional study was conducted to investigate the associations among professional identity, sleep quality, and anxiety symptoms in emergency department (ED) nurses. In addition, the potential mediating role of sleep quality in the relationship between professional identity and anxiety was explored.

### 2.2 Study population

The study was carried out among registered nurses working in the emergency departments of a tertiary teaching hospital in China between March and June 2024. Participants were recruited using convenience sampling. The inclusion criteria were as follows:

(1) Registered nurses with a valid nursing license;
(2) Working in the emergency department for at least 6 months; and
(3) Voluntary participation with informed consent provided.

Exclusion criteria included:

(1) Nursing interns or students;
(2) Nurses on long-term leave or temporarily transferred to other departments; and
(3) Individuals with a prior diagnosis of severe psychiatric or sleep disorders.

A total of 179 valid questionnaires were collected and included in the final analysis.

### 2.3 Data collection

Data were collected anonymously through a self-administered questionnaire. Prior to the formal survey, all investigators received standardized training to ensure consistency in data collection. After being informed of the study purpose and procedures, participants completed the questionnaire independently, with an average completion time of approximately 10–15 min. The questionnaire consisted of four parts: demographic information, the Professional Identity Scale for Nurses (PISN), the Pittsburgh Sleep Quality Index (PSQI), and the Generalized Anxiety Disorder-7 (GAD-7) scale. Each participant ID was permitted to submit the questionnaire only once, and all personal information was kept strictly confidential. To ensure data quality, questionnaires with more than 10% missing data or those exhibiting obvious response patterns were excluded from the final analysis.

### 2.4 Measurement instruments

#### 2.4.1 Professional Identity Scale for Nurses (PISN)

Professional identity was measured using the Professional Identity Scale for Nurses developed by Liu et al. (14). The scale consists of 30 items covering five dimensions: professional identity evaluation, professional social support, professional social proficiency, dealing with professional frustration, and professional self-reflection. Each item is rated on a 5-point Likert scale (1 = strongly disagree to 5 = strongly agree), yielding a total score ranging from 30 to 150, with higher scores reflecting a stronger professional identity.

According to the established norm (14), total scores ≤ 90 indicate low, 91–120 indicate medium, and >120 indicate high professional identity. In this study, all item codings (including reverse-scored items) were re-checked before analysis. The scale demonstrated excellent internal consistency, with a Cronbach's α of 0.938; subscale α coefficients ranged from 0.876 to 0.913, consistent with previous validation studies in Chinese nurses.

#### 2.4.2 Pittsburgh Sleep Quality Index (PSQI)

Sleep quality was assessed using the Chinese version of the Pittsburgh Sleep Quality Index (PSQI), a widely used instrument for evaluating subjective sleep quality over the past month. The PSQI contains 19 self-reported items, which yield seven components: sleep duration, sleep latency, sleep disturbances, subjective sleep quality, sleep efficiency, use of sleep medication, and daytime dysfunction. Component scores range from 0 (no difficulty) to 3 (severe difficulty) and are summed to obtain a global score ranging from 0 to 21(15).

Consistent with Chinese population studies, a global PSQI score of ≥7 was used to define poor sleep quality or sleep disorder. Higher scores indicate poorer sleep. In this study, the internal consistency of the PSQI was good (Cronbach's α = 0.821).

#### 2.4.3 Generalized Anxiety Disorder 7-item scale (GAD-7)

Anxiety symptoms were measured using the Generalized Anxiety Disorder-7 (GAD-7) scale developed by Spitzer et al. (16). The scale consists of seven items, each rated from 0 (not at all) to 3 (nearly every day), producing a total score ranging from 0 to 21.

Following established cutoffs, scores of 5, 10, and 15 indicate mild, moderate, and severe symptoms, respectively. For this study, a score of ≥5 was used as the cutoff to identify the presence of anxiety symptoms, consistent with prior research and the analytic plan for the present dataset. The internal consistency of the scale in this sample was excellent (Cronbach's α = 0.915).

### 2.5 Statistical analysis

All statistical analyses were performed using SPSS (version 26.0; IBM Corp., Armonk, NY, USA) and R (version 4.1.2; R Foundation for Statistical Computing, Vienna, Austria).

Categorical variables were presented as counts and percentages, while continuous variables were expressed as mean ± standard deviation (SD) for normally distributed data or as median with interquartile range (IQR) for non-normally distributed data. Group differences between nurses with and without anxiety were analyzed using independent-samples *t*-tests, Mann–Whitney *U* tests, or Chi-square tests, as appropriate.

To test whether sleep quality mediated the relationship between professional identity and anxiety, a mediation analysis was conducted using the PROCESS macro in R with 5,000 bootstrapped resamples.

The indirect effect was considered statistically significant when the 95% bias-corrected confidence interval (CI) did not include zero. All statistical tests were two-tailed, and a *p*-value < 0.05 was considered statistically significant.

Given the cross-sectional nature of the data, the mediation analysis was conducted to explore statistical associations rather than to infer causal mechanisms.

### 2.6 Ethical considerations

This study was approved by the Ethics Committee of West China Hospital (No. 2023/177). Written informed consent was obtained from all participants prior to data collection.

The study adhered to the ethical standards of the Declaration of Helsinki, and participants' anonymity and confidentiality were strictly maintained throughout the research process.

## 3 Results

### 3.1 Demographic and clinical characteristics of participants

A total of 179 emergency nurses participated in this study. Based on the GAD-7 cutoff score of 5, 110 nurses (61.5%) were classified as non-anxious and 69 (38.5%) were classified as anxious. The demographic characteristics of the two groups are summarized in Table 1. No significant differences were found between the groups in terms of age, gender, marital status, education, child status, professional title, or years of work experience (all *p* > 0.05), indicating comparability across these variables.

**Table 1:** Demographic characteristics and main variables of participants (*N* = 179).

**Variables**	**Total (*n* = 179)**	**Non-anxiety group (*n* = 110)**	**Anxiety group (*n* = 69)**	**Test statistic**	***p*-value**
**Age (years)**, ***n*** **(%)**					
18–45	156 (87.2%)	97 (88.2%)	59 (85.5%)	*X*^2^ = 0.150	0.699
46–69	23 (12.8%)	13 (11.8%)	10 (14.5%)		
**Gender**, ***n*** **(%)**					
Male	13 (7.3%)	10 (9.1%)	3 (4.3%)	*X* ^2^ = 1.230	0.267
Female	166 (92.7%)	100 (90.9%)	66 (95.7%)		
**Marital status**, ***n*** **(%)**					
Single	48 (26.8%)	28 (25.5%)	20 (29.0%)	*X*^2^ = 0.100	0.752
Married	131 (73.2%)	82 (74.5%)	49 (71.0%)		
**Education level**, ***n*** **(%)**					
College	23 (12.8%)	11 (10.0%)	12 (17.4%)	*X*^2^ = 4.120	0.127
Bachelor	142 (79.3%)	91 (82.7%)	51 (73.9%)		
Master or above	14 (7.8%)	8 (7.3%)	6 (8.7%)		
**Child status**					
No child	63 (35.2%)	37 (33.6%)	26 (37.7%)	*X*^2^ = 0.320	0.571
Have child	116 (64.8%)	73 (66.4%)	43 (62.3%)		
**Title**					
Junior or low	112 (62.6%)	71 (64.5%)	41 (59.4%)	*X*^2^ = 0.890	0.641
Intermedia	49 (27.4%)	28 (25.5%)	21 (30.4%)		
Senior	18 (10.1%)	11 (10.0%)	7 (10.1%)		
**Years of working experience**, ***n*** **(%)**					
< 5 years	4 (2.2%)	3 (2.7%)	1 (1.4%)	*X*^2^ = 0.580	0.748
6–10 years	9 (5.0%)	5 (4.5%)	4 (5.8%)		
>11 years	166 (92.7%)	102 (92.7%)	64 (92.8%)		
Professional identity scores, Median (IRQ)	112.0 (100.0–123.0)	113.0 (101.0–124.0)	110.0 (98.0–121.0)	*U* = 3,297, *z* = −1.230	0.219
PSQI score, Median (IRQ)	8.0 (6.0–11.0)	7.0 (5.0–9.0)	10.0 (8.0–13.0)	*U* = 2,156, *z* = −5.120	< 0.001

Continuous variables are presented as mean ± SD or median (IQR); categorical variables as n (%).

Tests: χ^2^ = chi-square test; Mann–Whitney U test reported with *U, z* and *p*.

Non-anxiety: GAD-7 < 5 (*n* = 110); Anxiety: GAD-7 ≥5 (*n* = 69).

Significant differences, however, were observed in the key study variables. Nurses in the anxiety group reported significantly poorer sleep quality, as indicated by higher PSQI scores (Median = 10.0, IQR: 8.0–13.0) compared to the non-anxiety group (Median = 7.0, IQR: 5.0–9.0; *U* = 2,156.000, *z* = −5.120, *p* < 0.001). Although professional identity scores were lower in the anxiety group (Median = 110.0, IQR: 98.0–121.0) than in the non-anxiety group (Median = 113.0, IQR: 101.0–124.0), this difference did not reach statistical significance (*U* = 3,297.000, *z* = −1.230, *p* = 0.219). Although the group difference in professional identity did not reach statistical significance, professional identity was treated as a continuous variable in regression and mediation analyses. Continuous-variable modeling is more sensitive to subtle linear associations, which may explain why professional identity still showed a significant predictive effect on anxiety.

### 3.2 Bivariate analysis of professional identity and sleep quality

As detailed in Table 2, the prevalence of sleep disorder (PSQI ≥ 7) was markedly higher in the anxiety group (63/69, 91.3%) than in the non-anxiety group (55/110, 50.0%; χ^2^ = 34.560, *p* < 0.001). This result underscores a strong association between the presence of anxiety symptoms and poor sleep quality among emergency nurses.

**Table 2:** Comparison of professional identity and sleep quality by anxiety status among emergency nurses (*N* = 179).

**Variables**	**Total (*n* = 179)**	**Non-anxiety group (*n* = 110)**	**Anxiety group (*n* = 69)**	**Test statistic**	***p*-value**
Professional identity score, Median (IQR)	112.0 (100.0–123.0)	113.0 (101.0–124.0)	110.0 (98.0–121.0)	*U* = 3,295, *z* = −1.230	0.219
Sleep disorder (P ≥ 7), *n* (%)	118 (65.9%)	55 (50.0%)	63 (91.3%)	*X*^2^ = 34.560	< 0.001

PSQI: sleep disorder defined as PSQI ≥ 7.

Statistical tests: Mann–Whitney U test for continuous variables, chi-square test for categorical variables.

### 3.3 Mediation analysis of sleep quality

**Figure 1:**
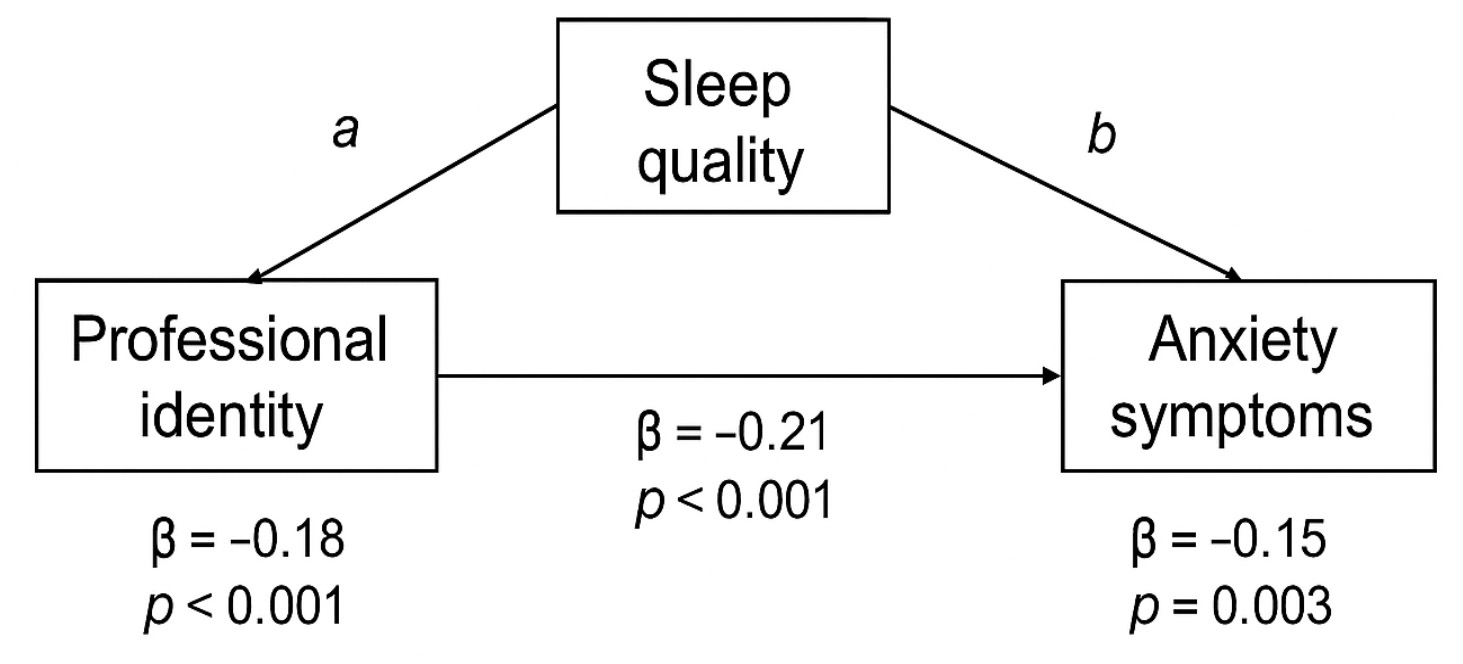
Mediating role of sleep quality in the relationship between professional identity and anxiety symptoms.

A mediation analysis with 5,000 bootstrapped samples was conducted to examine whether sleep quality mediates the relationship between professional identity and anxiety (Figure 1).

Professional identity was negatively associated with anxiety [total effect, c path: β = −0.21, *p* < 0.001, 95% CI (−0.31, −0.11)] and positively associated with better sleep quality [a path: β = −0.18, *p* < 0.001, 95% CI (−0.26, −0.10)]. Sleep quality, in turn, was positively associated with anxiety [b path: β = 0.32, *p* < 0.001, 95% CI (0.18, 0.46)] (Tables 3, 4).

**Table 3:** Pearson correlation matrix of key variables (*N* = 179).

**Variable**	**1**.	**2**.	**3**.	**4**.	**5**.	**6**.
1. Professional identity total	1					
2. PSQI total score	−0.31^***^	1				
3. GAD-7 total score	−0.27^***^	0.58^***^	1			
4. Age	0.08	−0.05	−0.04	1		
5. Years of experience	0.11	−0.07	−0.06	0.89^***^	1	
6. Gender^#^	0.09	−0.12	−0.10	0.15^*^	0.13	1

*N* = 179.

PSQI, Pittsburgh Sleep Quality Index; GAD-7, Generalized Anxiety Disorder 7-item Scale.

^#^Point-biserial correlation.

^*^p < 0.05, ^***^p < 0.001.

**Table 4:** Hierarchical regression analysis predicting anxiety symptoms (GAD-7).

**Predictor**	**Model 1**	**Model 2**	**Model 3**
**Step 1: demographic variables**			
Age	−0.08 (0.06)	−0.07 (0.05)	−0.06 (0.05)
Gender	−0.11 (0.42)	−0.09 (0.38)	−0.07 (0.35)
Years of experience	0.05 (0.07)	0.04 (0.06)	0.03 (0.06)
**Step 2: Professional identity**			
Professional identity total	–	−0.21^***^ (0.03)	−0.15^**^ (0.03)
**Step 3: Sleep quality**			
PSQI total score	–	–	0.32^***^ (0.07)
**Model statistics**			
*R* ^2^	0.018	0.063	0.185
Δ*R*^2^	0.018	0.045^***^	0.122^***^
*F*-value	1.05	3.78^**^	8.92^***^

Values represent standardized regression coefficients β (standard errors).

^**^p < 0.01, ^***^p < 0.001.

After accounting for sleep quality, the direct effect of professional identity on anxiety remained significant but was attenuated [c' path: β = −0.15, *p* = 0.003, 95% CI (−0.25, −0.05)]. The bootstrapped indirect effect was significant [a × b = −0.06, 95% CI (−0.10, −0.02)], indicating that sleep quality partially mediates the relationship between professional identity and anxiety, accounting for 28.6% of the total effect (Table 5).

**Table 5:** Mediation analysis of sleep quality in the relationship between professional identity and anxiety.

**Effect type**	**Effect value**	**SE**	**95% CI bootstrap**	***p*-value**
Total effect (c path)	−0.21	0.05	(−0.31, −0.11)	< 0.001
**Professional identity**→**Anxiety**				
Direct effect (c' path)	−0.15	0.05	(−0.25, −0.05)	0.003
**Professional identity**→**Anxiety**				
Indirect effect (a × b path)	−0.06	0.02	(−0.10, −0.02)	Significant
**Professional identity**→**Sleep quality**→**Anxiety**				
**Path coefficients**				
a path: Professional identity → Sleep Quality	−0.18	0.04	(−0.26, −0.10)	< 0.001
b path: Sleep quality → Anxiety	0.32	0.07	(0.18, 0.46)	< 0.001
Proportion mediated	28.6%	–	–	–

*N* = 179. Analysis conducted with 5,000 bootstrap samples. CI, confidence interval. Proportion mediated = Indirect effect/Total effect = 0.06/0.21 = 28.6%.

These mediation findings represent associative patterns among professional identity, sleep quality, and anxiety, and should not be interpreted as evidence of causal relationships.

## 4 Discussion

This cross-sectional study examined the relationships among professional identity, sleep quality, and anxiety symptoms in Chinese emergency nurses, and further explored whether sleep quality mediated the association between professional identity and anxiety. Several important findings emerged. Given the cross-sectional design, all interpretations in this section should be understood as reflecting statistical associations rather than causal processes.

### 4.1 Key findings and interpretation

First, the prevalence of anxiety among emergency nurses in this study was 38.5%, which is higher than that reported in general nursing populations. This aligns with previous research showing that emergency nurses are particularly vulnerable to psychological distress due to the high-intensity, unpredictable, and often traumatic nature of emergency care (17, 18). Interestingly, demographic and occupational factors—including age, gender, marital status, education level, professional title, and years of experience—were not significantly associated with anxiety. This suggests that work-related psychological and behavioral factors may play a larger role than personal background characteristics in determining anxiety risk (19).

Second, poor sleep quality was strongly associated with anxiety. Nurses with anxiety reported significantly worse sleep and were nearly twice as likely to have sleep disorders compared with those without anxiety. The strong positive correlation between PSQI scores and anxiety supports the notion that sleep disruption is a major determinant of mental health among shift-working nurses (9, 20). Emergency nurses often face rotating shifts and night duties, which contribute to occupational stress and are associated with poorer sleep quality, ultimately increasing susceptibility to anxiety (21).

Third, professional identity showed significant negative associations with both sleep quality and anxiety symptoms. Nurses with stronger professional identity tended to report better sleep and fewer anxiety symptoms. This finding is consistent with the Conservation of Resources theory, which posits that individuals seek to acquire and protect valuable resources. Professional identity can serve as a key psychological resource: nurses with a strong sense of professional purpose, competence, and belonging may cope more effectively with stress, maintain better emotional regulation, and experience fewer anxiety symptoms (22, 23). However, although professional identity showed a statistically significant association with anxiety in continuous-variable analyses, the absence of a significant group difference suggests that its effect is modest. Therefore, professional identity should be interpreted as a potential protective factor rather than a dominant determinant of anxiety among emergency nurses.

The most significant contribution of this study is the elucidation of the mediating role of sleep quality. The significant indirect effect (accounting for 28.6% of the total effect) provides a plausible mechanistic explanation for how professional identity influences mental health. A strong professional identity, characterized by a sense of purpose, competence, and belonging, may serve as a psychological buffer (22). It potentially helps nurses to process work-related stressors more effectively, reducing cognitive intrusion and physiological hyperarousal at night, thereby preserving sleep quality. Better sleep, in turn, enhances emotional stability and cognitive capacity to cope with next-day challenges, ultimately reducing anxiety levels (24). This pathway underscores that the benefits of professional identity extend beyond immediate job satisfaction, influencing fundamental restorative processes like sleep. Our mediation analysis indicated that the independent variable was statistically associated with the outcome variable through the mediator. However, given the cross-sectional nature of the data, these findings should be interpreted as associations rather than causal effects. Confirming causal pathways would require longitudinal or experimental studies that establish temporal ordering and rule out alternative explanations. Consequently, the observed mediation effect represents an indirect relationship in the data rather than a verified causal mechanism.

Within the Chinese healthcare context, heavy workloads, frequent night shifts, and strong expectations of professional commitment may place substantial psychological strain on emergency nurses, which may partly account for the observed associations among professional identity, sleep quality, and anxiety.

### 4.2 Implications for practice and policy

Our findings have important practical implications for hospital administrators and nurse leaders.

1. Intervention development: interventions aimed at reducing anxiety among emergency nurses should adopt a dual-pronged approach. Alongside traditional psychological support, programs specifically designed to strengthen professional identity—such as mentorship, reflective practice groups, and continuing education that reinforces the value and impact of nursing care—should be implemented.
2. Sleep health promotion: healthcare organizations should recognize sleep health as a critical component of employee wellbeing. Institutional support could include providing restful break areas, offering workshops on sleep hygiene tailored for shift workers, and fostering a culture that discourages excessive overtime, which directly encroaches on sleep time.
3. Preventive strategy: by bolstering professional identity and protecting sleep quality, institutions may not only alleviate existing anxiety but also build long-term resilience within their nursing workforce, potentially mitigating burnout and turnover.

### 4.3 Limitations and future research

Several limitations of this study should be acknowledged. First, the cross-sectional design precludes any definitive conclusions about causality. While our mediation model is theoretically grounded, the temporal relationships between professional identity, sleep quality, and anxiety need to be verified through longitudinal or experimental studies. Second, the data were collected through self-report measures, which may be subject to recall bias and social desirability bias. Future research could incorporate objective measures of sleep, such as actigraphy, to complement self-reported data. Third, the study was conducted in a single cultural context (China), and the generalizability of the findings to other healthcare systems and cultures requires further investigation. Finally, although we controlled for several key demographic and occupational variables, other potential confounding factors, such as specific workplace stressors or personality traits, were not assessed.

Future research should focus on:

- Longitudinal studies to establish the temporal precedence of these variables.
- Designing and evaluating interventions that simultaneously target professional identity and sleep hygiene.
- Exploring other potential mediators in this relationship, such as coping styles or social support.

## 5 Conclusion

In summary, this study highlights the complex interplay between professional identity, sleep quality, and anxiety among emergency nurses. Strong professional identity is associated with better sleep and lower anxiety, with sleep partially mediating this relationship. Interventions that foster professional identity and promote sleep health may provide an effective approach to supporting the mental wellbeing of emergency nurses, ultimately contributing to the sustainability of high-quality emergency care.

## Data Availability

The raw data supporting the conclusions of this article will be made available by the authors, without undue reservation.
